# Biomechanical impacts of 3D arch-support insoles on countermovement jumps: a statistical parametric mapping analysis

**DOI:** 10.3389/fbioe.2025.1624892

**Published:** 2025-08-26

**Authors:** Qiu-qiong Shi, Kit-lun Yick, Rui-feng Huang, Chu-hao Li, Jin long Wu

**Affiliations:** ^1^ School of Fashion and Textiles, The Hong Kong Polytechnic University, Hong Kong, China; ^2^ Research Institute for Sports Science and Technology, Hong Kong, China; ^3^ Engineering Research Center of Sports Health Intelligent Equipment of Hubei Province, Wuhan Sports University, Wuhan, China; ^4^ College of Physical Education, Southwest University, Chongqing, China

**Keywords:** arch-support, 3D insole, orthoses, statistical parametric mapping, jumping

## Abstract

**Objective:**

Despite the widespread use of arch-support insoles in sports, their time-dependent biomechanical effects on dynamic movements like countermovement jumps (CMJs) remain poorly understood. This study investigated the biomechanical impacts of three-dimensional (3D) arch-support insoles with varying degrees of stiffness on CMJs by using a statistical parametric mapping (SPM) analysis.

**Design:**

Randomized crossover study.

**Method:**

Twelve active male university students tested three different polyurethane 3D arch-support insoles (i.e., soft, semi-rigid, and rigid insoles). A total of 16 reflective markers were placed on the lower limbs of the participants according to the Vicon Plug-in Gait marker set protocols. The lower limb kinematics and kinetics were captured by using two synchronized force plates and an eight-camera motion analysis system. SPM was used to statistically compare the biomechanical changes across the different 3D insoles during six continuous key phases of CMJs.

**Results:**

With the 3D arch-support insoles donned, supra-threshold clusters of the ankle kinematics in the sagittal and frontal planes exceeded the critical thresholds during propulsion-flight (*p* = 0.022) and the landing (*p* = 0.033). Ankle moment in the transverse direction exceeded the critical threshold of 6.46 during propulsion (*p* = 0.038) and landing (*p* < 0.001). The critical threshold of 6.555 was exceeded for propulsion (*p* = 0.050) and landing (*p* < 0.001) with supra-threshold clusters for the force in the frontal plane of the knee. Ankle force in the transverse direction showed that the supra-threshold clusters exceeded the critical threshold during weighing-unweighting (*p* < 0.001), and early landing (*p* = 0.007).

**Conclusion:**

Rigid and semi-rigid 3D arch-support insoles significantly altered the biomechanics of the ankle joint, primarily in the frontal and transverse planes during propulsion-flight, and the landing phases. The rigid 3D insole most effectively enhanced ankle joint stability, which is crucial for maintaining balance and preventing injuries. SPM provided a time-dependent analysis of the biomechanical impacts during CMJ.

## 1 Introduction

Countermovement jumps (CMJs) are a very common jumping task in a number of different sports, including basketball, volleyball, and badminton. During CMJs, the feet and lower extremities are subjected to extremely high ground reaction forces (GRFs) (Tillman et al., 2003), which can reach up to nine times the body weight of an individual during landing from a jump (Abdelkrim et al., 2007). Such forces contribute to a significant risk of injury, particularly in sports like basketball, in which around 58% of all injuries occur after landing from a jump (Gray et al., 1985). These forces pose a significant risk to the musculoskeletal system, particularly the ankle, knee, and hip joints. The knee joint is particularly vulnerable, which accounts for 75% of the lower extremity injuries in basketball (Gray et al., 1985), and 59% in volleyball (Gerberich et al., 1987), with anterior cruciate ligament tears being particularly common due to excessive valgus loading (Sadeqi et al., 2023). The ability of the musculoskeletal system to modulate these high-impact forces is critical for preventing injuries. For example, the larger flexion angles of the knee and hip joints during landing may increase shock absorption and reduce joint reaction force, which will prevent lower limb injury (Lee et al., 2018). Similarly, increased ankle dorsiflexion and knee flexion, reduced hip abduction and adduction moments, are associated with lower risk of injury during CMJs (Fong et al., 2011; Sadeqi et al., 2023).

Three-dimensional (3D) arch-support insoles have been widely used to optimize movement biomechanics and prevent lower extremity injuries (Jor et al., 2025). These devices increase the contact area and pressure distribution at the medial longitudinal arch of the foot (Shi QQ. et al., 2022a; Shi Q-Q. et al., 2022b), thus enhancing postural stability and stimulating the sensory receptors on the plantar surface of the foot during movement (Jenkins and Raedeke, 2006). Variation in insole stiffness can alter the mechanical loading transmitted to the musculoskeletal system during impact, potentially influencing movement biomechanics and injury risk. Many previous studies investigated that modifying midsole stiffness of footwear could alter ankle motion and facilitate energy return to reduce injury risk during jumping (Taylor et al., 2019; Cigoja et al., 2020). Previous study found that softer insole may enhance jump height but increased vertical GRF (vGRF) loading rate, while the vGRF loading rate decreased significantly by using hard insole (Alirezaei Noghondar and Bressel, 2017). Studies have shown that 3D arch-support insoles can reduce peak ankle plantarflexion and eversion moments (Wang et al., 2020), decrease ankle inversion moments (Lam et al., 2022), and limit hip internal rotation (Jenkins et al., 2009) and adduction (Joseph et al., 2010) during landing tasks. However, other studies have reported that arch-support insoles may increase peak GRF values (Lam et al., 2022), peak knee adduction moments, and knee flexion angular velocity (Yue et al., 2022), as well as increase plantar forces, plantar pressure, and the maximum ankle inversion angle (Yu et al., 2007), thus suggesting a potential increased risk of injury. These biomechanical changes could potentially increase injury risk by altering normal joint loading patterns and potentially compromising stability during movement (Yue et al., 2022; Yu et al., 2007). Additionally, study report no significant effects of arch-support insoles on lower limb coordination during jumping (Arastoo et al., 2014; Noghondar and Yazdi, 2017). The ongoing controversy regarding the biomechanical effects of arch-support insoles during jumping and landing tasks needs further investigation, particularly given their clinical implications for injury prevention and performance optimization in athletes.

A key limitation of previous studies is their reliance on discrete parameters (e.g., means, peaks and timing) to examine the performance of arch-support insoles. Discrete parameters often fail to capture the full complexity of time series, such as the shape and pattern of kinematic and kinetic curves, thus limiting their ability to detect differences between conditions or populations. This limited analytical approach could lead to overestimating statistical significance, even in the absence of true differences exists between conditions or individuals (Moisan et al., 2021). To address this limitation, one-dimensional analysis methods, such as statistical parametric mapping (SPM), mitigate this risk by enabling comparisons of biomechanical outcomes for each percentage of a cyclic task without requiring multiple statistical corrections (Friston, 2003). This approach not only reduces the risk of inflated false positive rates but also provides a more intuitive and visually interpretable representation of the data (Serrien et al., 2019). By preserving the continuity of the time series, SPM enables researchers to identify subtle but meaningful differences that discrete parameters might otherwise overlook.

The maneuver of CMJs consists of distinct phases, including weighing, unweighting, braking, propulsion, flight, and landing (McMahon et al., 2018), each of which involves complex interactions between the kinematic and kinetic variables. For example, braking phrase called as the stretching phase, while propulsion phase is defined as the push-off phase whereby individuals forcefully extend their hips, knees, and ankles to propel their body vertically, while the flight phase is defined as departing from the force platform with the intention of attaining maximum jump height (McMahon et al., 2018). The propulsion phase is critical in determining CMJ performance, as it directly influences the jump height, power output, and movement efficiency. SPM has been suggested as an effective method for analyzing such one-dimensional data, and offering a more comprehensive understanding of movement dynamics compared to traditional discrete parameter analyses (Serrien et al., 2019; Nüesch et al., 2019; Huang et al., 2025). Despite its advantages, no previous studies have utilized SPM to investigate the effects of arch-support insoles of varying stiffness on kinematic and kinetic time series during CMJs. This study aims to fill this research gap by using SPM to provide a comprehensive, time-continuous analysis of the biomechanical changes across the entire CMJ cycle. Previous studies have predominantly focused on discrete peak values (e.g., maximal knee flexion at propulsion or ground reaction forces at landing), neglecting transitional phases (e.g., early braking or mid-landing) where insole stiffness may critically alter movement strategies. By leveraging SPM’s ability to detect time-dependent effects without arbitrary phase segmentation, this research offers novel insights into how arch-support insoles with various stiffnesses influence the lower limb biomechanical changes throughout the entire CMJ cycle. We hypothesized that SPM would reveal time-dependent biomechanical effects of insole stiffness during transitional CMJ phases (e.g., early braking, late-landing) that were undetectable through traditional discrete parameter analysis.

## 2 Materials and methods

### 2.1 Participants

Twelve active male university students (age: (mean ± SD) 26.8 ± 2.3 years old; height: 171.8 ± 4.6 cm; body weight: 67.0 ± 9.1 kg) with 6.0 ± 4.2 years of training experience were recruited, including 8 badminton, 2 basketball, 2 fitness and running specialists. The predicted recruitment sample size was calculated by using G*Power software, a large effect size of 0.8 was set for repeated measures analysis of variance (ANOVA), at least 10 participants were required with an α err probability of 0.05, and power level of 0.8 (Shi QQ. et al., 2022a; Shi Q-Q. et al., 2022b). The exclusion criteria included: (1) the presence of any injury, illness, or medical condition identified via the Physical Activity Readiness Questionnaire (PAR-Q), and (2) failure to meet the physical training requirements, (i.e., ≥ 2 years of training history, or ≤ 1 h/session with 2 sessions/week in the past 2 years). They were asked to sign a consent form prior to the experiment after given a briefing on the study. Human subject ethics approval for this study was granted by the Institutional Review Board of the University Ethics Committee (HSEARS20240206005). Each participant visited the laboratory once for testing. They were required to refrain from drinking caffeine or taking stimulants, and no physical training for 24 h before they participated in the tests.

### 2.2 Arch-support insoles

**FIGURE 1:**
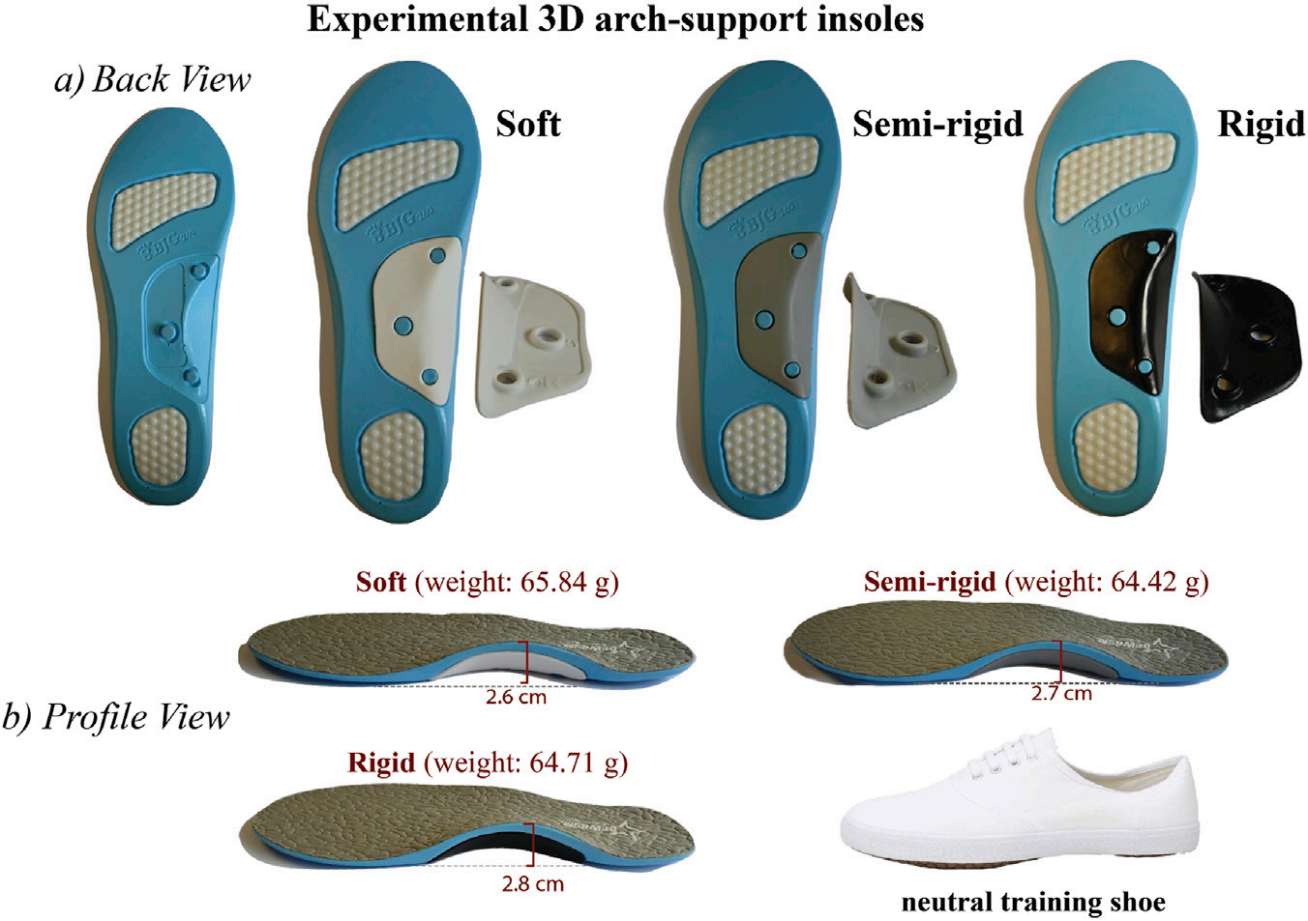
Prefabricated 3D arch-support insoles and neutral shoes used in study. **(a)** back view, and **(b)** profile view.

Adjustable 3D polyurethane insoles (CPWGSM, United States of America) with a prefabricated arch-support that has three levels of stiffness, including soft, semi-rigid, and rigid, are used in this study (see Figure 1). The information for the stiffness level is provided by the manufacturer. Due to the variations in stiffness, the 3D arch-support insoles have an average difference of 3.75% (1 mm) in arch height and 2% (0.95 g) in weight. The participants wore a commonly available type of training shoe (Double Star, China) in their respective foot size with each type of 3D arch-support insole or without any insole (control). To minimize the order effect, conditions were randomized using a balanced Latin square design (Bradley, 1958), ensuring each stiffness level appeared equally in each position across participants. Additionally, sufficient rest intervals (≥15 min) were enforced between trials to mitigate fatigue or carryover effects.

### 2.3 Protocols

Prior to the data collection, the participants completed a 10-min warm-up session (LaPorta et al., 2013) and acclimatized to the testing conditions by practicing 3–5 trials of CMJs. Then a total of 16 reflective markers of 14 mm in diameter were placed on the lower limbs of the participants, according to the Vicon Plug-in Gait marker set protocols (Shi Q-Q. et al., 2022b; Kadaba et al., 1989). To ensure consistency and reliability, the same senior researcher attached the markers onto each participant.

During the CMJ trials, the participants would stand on the centre of the force plates with their hands on their iliac crest to minimize arm swing (Kajiwara et al., 2019). They were instructed to perform a CMJ and land back onto the center of the force plates, and maintain a neutral and quasi-static state for 3 s to ensure that the data were properly captured (Wikstrom et al., 2005). Trials were considered unsuccessful if the participant failed to remain stationary for the required 3 s or removed his hands from his iliac crest during the CMJ. To prevent fatigue, the participants were given a break of 30 s between trials, and a 15-min break was also given between the different orthosis conditions. Kinematic and kinetic data were collected by using two synchronized force plates with dimensions of 60 cm × 40 cm (AMTI, United States of America) and an eight-camera motion analysis system (VICON, Nexus 2.0 Inc., Oxford, United Kingdom) at a sampling frequency of 200 Hz. Five successful trials were recorded for each condition.

### 2.4 Phases of CMJ

**FIGURE 2:**
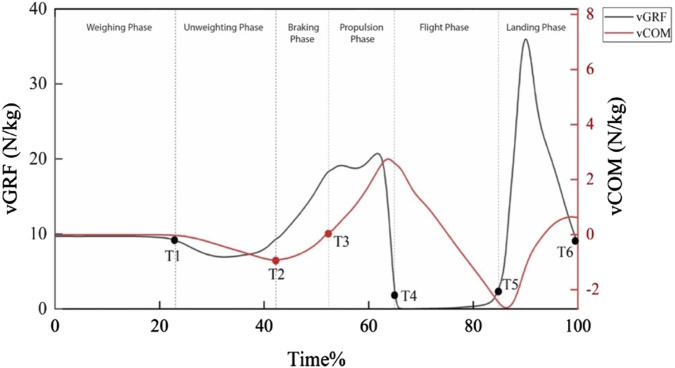
Phases during CMJs.

Based on the existing literature that have analyzed data on CMJs, the phases of the CMJs are defined according to six time points (McMahon et al., 2018) in Figure 2 (black curve indicates the vGRF during a CMJ (total time is 100%), and red curve indicates the velocity of the center of mass (vCOM) during a CMJ (total time is 100%)): (1) weighing phase, which is from T0 to T1, where T1 is the time when the vGRF is reduced by a threshold equal to 5 times the standard deviation of the initial weighing value; (2) unweighting phase, which is from T1 to T2, where T2 is the time when the vCOM meets the minimum value; (3) braking phase, which is from T2 to T3, where T3 is the time when vCOM increases to over 0 after T2; (4) propulsion phase, which is from T3 to T4, where T4 is the time when vGRF decreases to less than 10% of the initial weighing value; (5) flight phase, which is from T4 to T5 where T5 is the time when vGRF increases to greater than 10% of the initial weighing value; and (6) landing phase, which is from T5 to T6 where T6 is the time when the vGRF decreases to 95% of the initial weighing value.

### 2.5 Indicators

**FIGURE 3:**
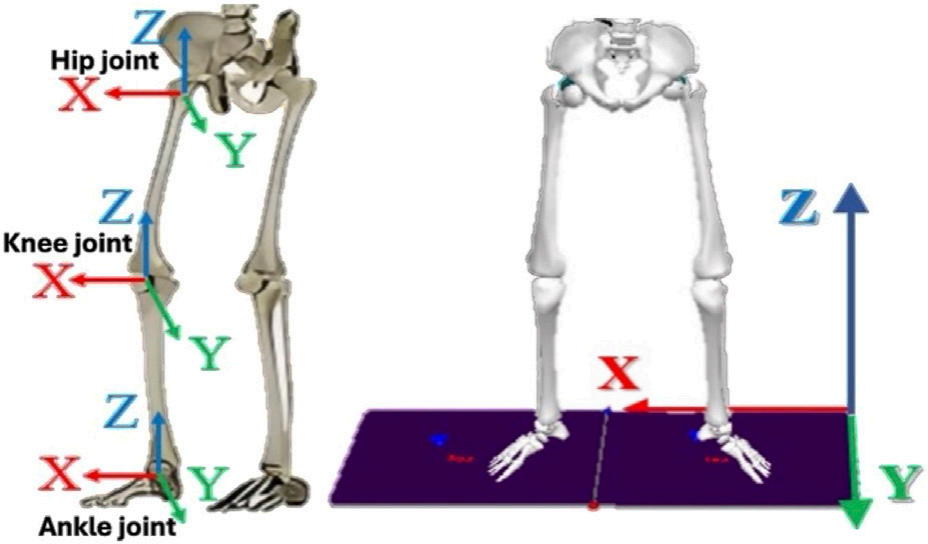
Schematic of joints and coordinate systems.

The kinematics of the participants was captured with the reflective markers. The ankle, knee, and hip joint angular displacements were calculated for each participant and compared based on with and without the different arch-support conditions when wearing neutral shoes. The kinetics data were calculated by using the vGRF during jumping. The ISB global joint coordinate system used for the calculations is presented in Figure 3. Joint angles, forces, and moments are expressed in three anatomical planes, including the sagittal, frontal, and transverse planes. Following the Plug-in Gait Reference Guide (Vicon Motion Systems Limited) (Plug‐in Gait Reference Guide, 2023), Supplementary Table S1 presents the biomechanical variables for the lower limb joints which were investigated on the three planes during CMJ cycles.

### 2.6 Data analysis

The three best jumps for each participant were selected based on maximal jump height, with proper landing (i.e., full foot contact and no balance loss). These trials were obtained through marker labeling, gap filling, and establishing a skeletal model of lower limbs to determine the kinematic and kinetic information in the Vicon software. All of the joint center trajectories were smoothed by using a recursive fourth-order low-pass Butterworth filter, with a cutoff frequency of 6 Hz selected through a residual analysis and visual inspection of the data (McErlain-Naylor et al., 2014). The moment and force parameters were normalized to the height and weight of the participants (Shi Q-Q. et al., 2022b). The dominant foot was determined by conducting a procedure during which the participant kicked a ball (Shi QQ. et al., 2022a). All of the participants used their right foot as their dominant foot.

### 2.7 Statistics

SPM analyzed the effect of insole condition (single factor: soft/semi-rigid/rigid/no 3D arch-support insole) on time-normalized biomechanical trajectories. Unlike factorial ANOVA, SPM does not discretize time into phases but instead identifies supra-threshold temporal regions where conditions differ significantly, accounting for temporal smoothness via random field theory (Pataky, 2010). Initially, the one-way repeated measures analysis of variance (ANOVA) was applied to the normalized time series to detect the overall differences among the experimental insole conditions. Where significant effects were identified (*p* < 0.05), *post hoc* paired *t*-tests were performed over the normalized time series to localize the differences between the specific orthotic configurations. Technical procedures were implemented in MATLAB (MathWorks Inc., United States of America) by using the spm1D package (v0.4) (Patak and y, 2012), which addresses the multiple-comparison problem inherent to time-series analyses. Further methodological details can be found in previous studies (Pataky, 2010; Penny et al., 2011).

The SPM analysis followed four sequential steps.

a. The test statistics (F- or t-values) were calculated at each time point within the normalized time series.
b. Then temporal smoothness was estimated by calculating the average temporal gradient of the residuals, a critical step for adjusting statistical thresholds with the random field theory to account for multiple comparisons.
c. The critical threshold value of the test statistic was determined, above which only 5% of the data would be expected to reach if the trajectory resulted from an equally smooth random process.
d. Finally, the probability of observing contiguous suprathreshold clusters, defined as the time intervals in which the test statistic exceeds the critical threshold, was evaluated to identify statistically significant temporal regions (De Ridder et al., 2013).

## 3 Results

### 3.1 Kinematic

#### 3.1.1 Jump height

Insole conditions had no significant main effect on jump height (*p* = 0.33), nor significance was found by *post hoc* analysis among insole conditions and control.

#### 3.1.2 Joint angles

**FIGURE 4:**
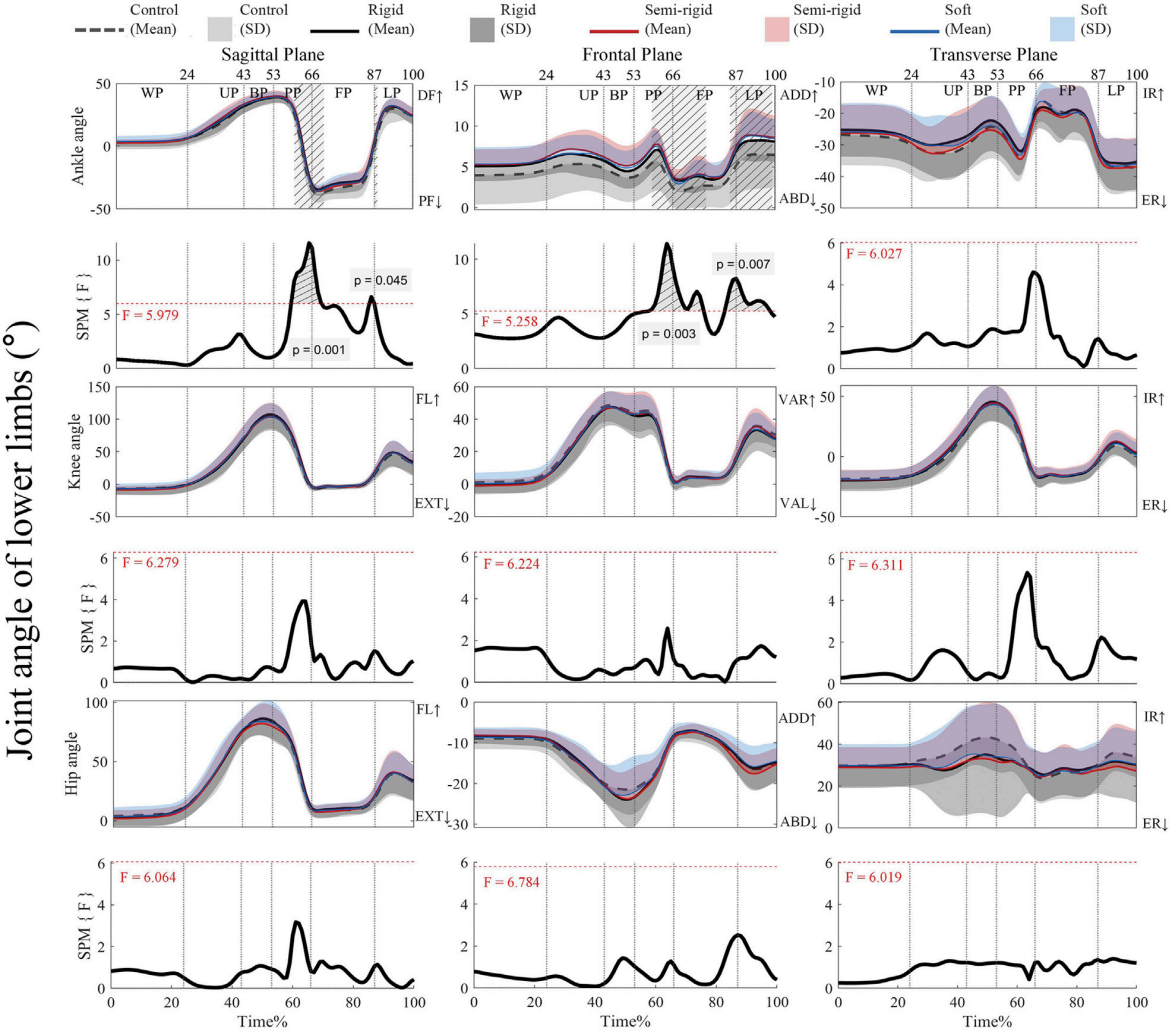
Mean (SD) patterns for lower limb joint angles with and without 3D arch-support insoles and time-dependent F-values of SPM. Light grey with stripes indicate regions with statistical difference. Red dashed line represents the critical threshold. WP, weighing phase; UP, unweighting phase; BP, braking phase; PP, propulsion phase; FP, flight phase; LP, landing phase; DF, dorsiflexion; PF, plantarflexion; ADD, adduction; ABD, abduction; IR, internal rotation; ER, external rotation; FL, flexion; EXT, extension; VAR, varus; VAL, valgus.

Figure 4 shows that the supra-threshold clusters in the sagittal plane of the ankle joint exceed the critical threshold of 5.979 during propulsion-flight (59%–69%) and early landing (86%–88%). Similarly, in the frontal plane of the ankle joint, the supra-threshold clusters (58%–76% and 84%–98%) exceed the critical threshold of 5.258, with variations observed across the 3D arch-support insoles. Supplementary Figures S1–S3 present the *post hoc* analysis with paired t-tests for the joint angles of the ankle, knee, and hip.

Supplementary Figure S1 shows that during propulsion, the critical threshold of 3.277 is exceeded (65%–66%) with a negative supra-threshold cluster (*p* = 0.040), thus indicating that ankle plantarflexion is significantly less negative by wearing a rigid 3D arch-support insole compared to the control. In the frontal plane, the negative supra-threshold clusters (66%–67%) exceed the critical threshold of −3.081, which shows that ankle abduction is significantly less negative with the use of the rigid 3D insole during flight (*p* = 0.039). On the other hand, the critical threshold of −3.071 is exceeded for propulsion-flight (63%–72%) and early landing (87%–91%) with negative supra-threshold clusters, which shows that ankle adduction during propulsion-flight (*p* = 0.022), and ankle adduction during landing (*p* = 0.033) are significantly more negative with the control condition than with the use of a semi-rigid 3D arch-support insole. There are no supra-threshold clusters that exceed the critical threshold for the other kinematic parameters.

### 3.2 Kinetics

#### 3.2.1 Joint moments

**FIGURE 5:**
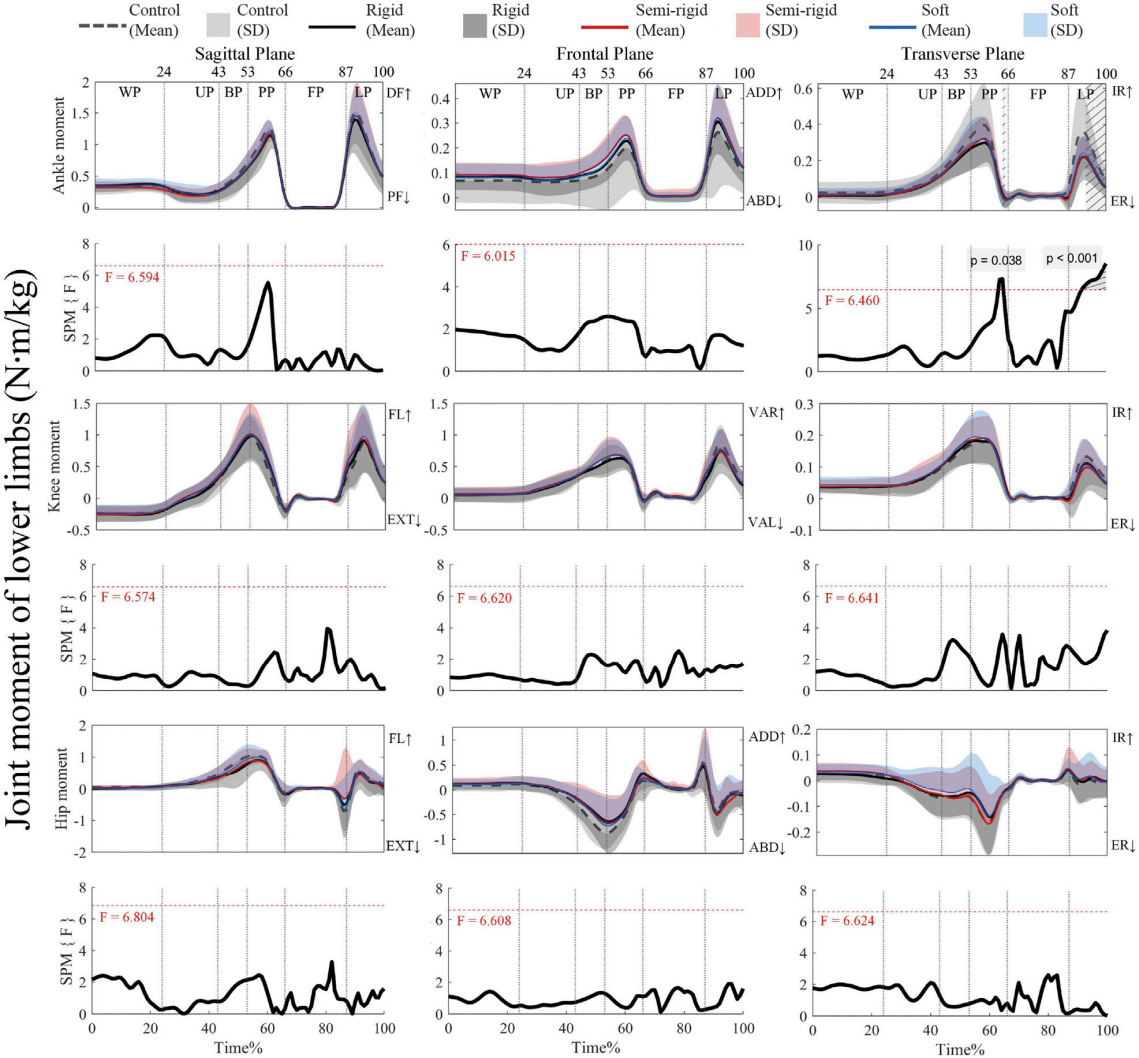
Mean (SD) patterns for lower limb joint moments with and without 3D arch-support insoles and time-dependent F-values of SPM (SPM {F}). Light grey with stripes represent regions with statistical difference. Red dashed line represents the critical threshold. WP, weighing phase; UP, unweighting phase; BP, braking phase; PP, propulsion phase; FP, flight phase; LP, landing phase; DF, dorsiflexion; PF, plantarflexion; ADD, adduction; ABD, abduction; IR, internal rotation; ER, external rotation; FL, flexion; EXT, extension; VAR, varus; VAL, valgus.

Figure 5 shows that the critical threshold of 6.46 is only exceeded during propulsion (64%–65%) and landing (92%–100%) with supra-threshold clusters (*p* = 0.038 (PP), and *p* < 0.001 (LP)) for the ankle transverse moment when using the 3D arch-support insoles. Supplementary Figures S4–S6 present the *post hoc* analysis with paired t-tests for the moments of the ankle, knee, and hip joints.

Supplementary Figure S4 shows that the critical threshold of 3.436 is exceeded for landing (91%–100%) with a positive supra-threshold cluster (*p* = 0.001), which means that the internal rotation moment of the ankle in the control condition is significantly more positive than with the use of a rigid 3D insole. On the other hand, the critical threshold of 3.457 is exceeded for landing (97%–100%) with positive supra-threshold clusters (*p* = 0.021), which shows that the internal rotation moment of the ankle is significantly less positive with a semi-rigid 3D arch-support insole. Finally, an analysis of the other joint moments show no supra-threshold clusters that exceed the critical threshold.

#### 3.2.2 Joint forces

**FIGURE 6:**
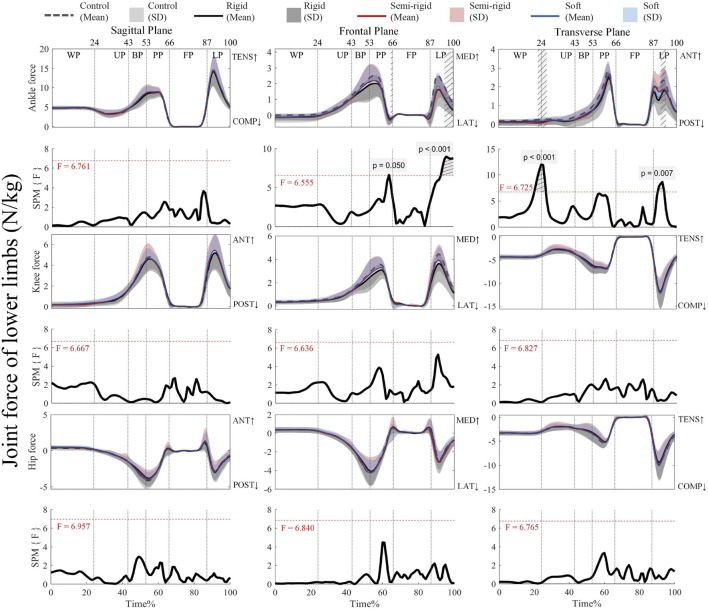
Mean (SD) patterns for lower limb joint forces with and without 3D arch-support insoles and time-dependent F-values of SPM (SPM {F}). Light grey with stripes indicate regions with statistical difference. Red dashed line represents the critical threshold. WP, weighing phase; UP, unweighting phase; BP, braking phase; PP, propulsion phase; FP, flight phase; LP, landing phase; ANT, anterior; POST, posterior; TENS, tension; COMP, compression.

Figure 6 shows that the critical threshold of 6.555 is exceeded for propulsion (65%–66%) and landing (94%–100%) with supra-threshold clusters (*p* = 0.050 (propulsion), and *p* < 0.001 (landing) for the ankle force in the frontal plane. On the other hand, the critical threshold of 6.725 is exceeded for weighing-unweighting (21%–26%) and landing (90%–93%) with supra-threshold clusters (*p* < 0.001 (weighing-unweighting), and *p* = 0.007 (landing) for the ankle force in the transverse plane with the 3D arch-support insoles. Supplementary Figures S7–S9 present the *post hoc* analysis with paired t-tests for the joint forces of the ankle, knee, and hip.

Supplementary Figure S7 shows that the positive supra-threshold clusters (94%–100%) exceed the critical threshold of 3.542, thus indicating that the medial force at the ankle with the control condition is significantly more positive than wearing a rigid 3D arch-support insole during landing (*p* < 0.001). In the transverse plane, the critical threshold of 3.547 is exceeded for landing (90%–91%) with positive supra-threshold clusters (*p* = 0.035), and the anterior force at the ankle is significantly more positive with the control than a rigid 3D arch-support insole. On the other hand, the critical threshold of 3.566 is exceeded for weighing-unweighting (21%–24%) with a positive supra-threshold cluster (*p* = 0.007), thus revealing that the anterior force at the ankle is significantly less positive with a semi-rigid 3D arch-support insole. Additionally, the positive supra-threshold clusters (90%–91%) exceed the critical threshold of 3.557, which shows that the anterior force at the ankle is significantly less positive with a soft 3D arch-support insole during landing (*p* = 0.040). No other joint force parameters have supra-threshold clusters that exceed the critical threshold.

#### 3.2.3 vGRF

**FIGURE 7:**
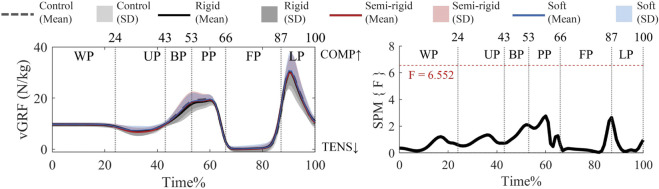
Mean (SD) patterns for vGRF with and without 3D arch-support insoles and time-dependent F-values of SPM (SPM {F}). Red dashed line represents the critical threshold. WP, weighing phase; UP, unweighting phase; BP, braking phase; PP, propulsion phase; FP, flight phase; LP, landing phase.

Figure 7 shows that there are no supra-threshold clusters that exceed the critical threshold for vGRF, which is in agreement with the results of the *post hoc* analysis with a paired-t test (Supplementary Figure S10).

## 4 Discussion

This study represents the first comprehensive investigation using SPM to analyze how 3D arch-support insoles of different stiffness levels affect biomechanics during CMJs. The key findings show that rigid and semi-rigid 3D arch-support insoles significantly alter the biomechanics of the ankle joint compared to the control (no 3D insole), particularly during propulsion-flight, and the landing phases, but no significant influence on the jump height, vGRF, and knee and hip joints. These results provide novel insights into the dynamic role of 3D arch-support insoles in modulating jump mechanics, thus addressing a gap in previous research work that is limited to zero-dimensional parameters.

### 4.1 Biomechanical effects of 3D arch-support insoles during propulsion–flight phase

Our findings showed that the 3D arch-support insoles significantly altered the ankle biomechanics during propulsion-flight phase of CMJ. The results are consistent with our hypothesis that some impactful changes failed to be detected by previous zero-dimensional data analysis. Specifically, the insole led to significantly reduced ankle plantarflexion at 59%–69% of CMJ, and increased ankle adduction at 58%–76% of CMJ compared to the control condition. The rigid 3D insole significantly decreased ankle plantarflexion at 65%–66% (propulsion), and increased ankle adduction at propulsion (66%–67%). The semi-rigid 3D insole significantly increased ankle adduction at propulsion-flight (63%–72%). These narrow time windows likely correspond to critical transition points in force generation, where the foot shifts from mid-stance to push-off, suggesting that 3D arch-support insoles most strongly modulate ankle mechanics during rapid force redirection. While brief, these intervals may reflect key instants where foot rigidity influences propulsion efficiency.

These kinematic changes aligned with functional role of ankle plantarflexion and adduction dominate the propulsive phase (Welte, 2020). The observed increase in ankle adduction may be attributed to the medial support provided by the insoles, which modifies frontal-plane motion. This finding partially agrees with Yu et al. (2007), who also reported increased ankle adduction with arch-support interventions during landing. Aligning with Ho et al. (2019) reported that no significant interactions between insoles and foot type for jump height, kinematic and kinetic variables in basketball players, but the ankle eversion was significantly reduced by using prefabricated insoles during CMJs take-off moment, suggesting that the medial support of 3D arch-support insoles primarily altered the frontal plane kinematics.

The windlass effect is a mechanism in the foot that occurs during the push-off phase of jumping, may explain the altered ankle kinematics. Ker et al. (1987) proved that the elastic properties of human’s foot arch is essential for windlass effect. During dynamic loading, the foot arch stores strain energy, facilitating the windlass effect. This energy-releasing mechanism complements the elastic action of the Achilles tendon during push-off (Ker et al., 1987). The 3D arch-support insoles could enhance this mechanism by reinforcing the medial longitudinal arch, thereby facilitating more effective force transfer during propulsion (Welte et al., 2018). Additionally, the semi-rigid insole’s faster compression (Figure 4) could optimize elastic recoil during weighting-unweighting (21%–24%), mirroring the energy-storing role of the arch described by Ker et al. (1987). This suggests that the insoles augment the foot’s natural spring-like function, facilitating the foot’s capability in storing strain energy and returning it in as elastic recoil, whilst improving push-off efficiency.

While the 3D arch-support insoles did not significantly affect knee and hip joint moments, they did reduce hip flexion and abduction moments during the braking-propulsion phase compared to the control. Excessive hip abduction can cause undesirable lateral shifts in center of pressure (Lee and Powers, 2014). The insoles appear to influence hip stability in both sagittal and frontal planes during this critical push-off phase by significantly modifying ankle biomechanics. Our study is the first to report increased ankle adduction during propulsion–flight with semi-rigid 3D insoles by SPM. This finding suggested that 3D insoles, especially the semi-rigid one, may optimize push-off efficiency by promoting faster energy return and reducing unnecessary motion. Future research should explore whether these biomechanical adjustments translate to improved jump performance (e.g., height or power) in athletic populations.

### 4.2 Biomechanical effects of 3D arch-support insoles during landing phase

Following the ankle kinematic changes, most of significant ankle kinetic alteration occurred during landing (90%–100%) by wearing 3D arch-support insoles. The rigid insole significantly reduced transverse ankle internal rotation moment during 91%–100% of CMJ, with reduced ankle anterior force at 90%–91% and ankle medial force during 94%–100%. Semi-rigid 3D arch-support insole significantly increased ankle adduction during early landing (87%–91%), with reduced ankle internal rotation moment during 97%–100% of CMJ. While soft insole significantly reduced ankle anterior force during 90%–100%. The results verified our previous hypothesis that significant ankle biomechanical changes occurred during landing, but not at the peak value moments.

Notably, excessive moments of internal rotation in the transverse plane have been reported to exacerbate anterior cruciate ligament strain (Lee and Shin, 2021). This reduction of the internal ankle rotation moments aligns that arch’s ligaments (e.g., plantar calcaneonavicular/spring ligament) resist flattening under load, limiting aberrant joint motion (Ker et al., 1987). The rigid and semi-rigid insoles may amplify this stabilizing effect, mitigating forces linked to anterior cruciate ligament strain (Lee and Powers, 2014) by reinforcing midfoot rigidity. Previous study claimed that the lateral and anterior forces peak earlier which is considered to be less functional stability (Caulfield and Garrett, 2004). The rigid 3D arch-support insole may enhance ankle joint stability in the frontal and transverse planes during the propulsion and landing phases (Figure 7). We found that the insoles have little effect on the vGRF, which aligns with the findings in Wang et al. (2020). Our finding is consistent with the understanding that stiffer arch-support insoles increase midfoot or rearfoot rigidity without substantially altering the vGRF during landing movement (Zhang et al., 2012). Differently from previous study, Lou et al. (2015) whose participants showed a significantly reduced peak moment of the internal ankle rotation with the use of arch-support insoles. Our study found that 3D insoles significantly influenced the ankle biomechanics from mid-landing until stabilization.

These observed biomechanical changes in the ankle joints are largely due to the changes in the alignment of the foot with the use of the rigid and semi-rigid 3D arch-support insoles in this study. These 3D insoles made with varying material stiffness are recommended to mainly redistribute plantar forces to offer shock absorption and better cushioning (Shi QQ. et al., 2022a; Shi Q-Q. et al., 2022b). The insoles’ materials (i.e., rigid, and semi-rigid) may complement this by augmenting energy dissipation during landing, as evidenced by reduced ankle anterior forces (90%–100%). This synergy between intrinsic (arch) and extrinsic (insole) mechanisms could explain the protective effects observed (Ker et al., 1987). For clinicians, arch-support insoles are a vital tool for managing painful foot and lower extremity pathologies, which prevent injuries, and optimize the biomechanics during sports and other weight-bearing activities (Werd et al., 2010). The rigid and semi-rigid 3D insoles effectively influenced ankle joint in the frontal and transverse planes during landing phases, which is crucial for maintaining balance and reduce impact forces during landing, thereby protecting against over-use injuries. In line with Alirezaei Noghondar and Bressel (2017), our findings suggest that the 3D insoles especially the rigid one modulate loading pattern, thus potentially reducing the risk of injury during jumping-landing tasks. However, very few studies have investigated the biomechanical changes by examining the different degrees of rigidity of 3D arch-support insoles on CMJs, so CMJ performance by using 3D insoles with different degrees of stiffness warrants further investigation.

### 4.3 Limitations

There are several limitations in this study. First, while the 3D arch-support insoles were designed to influence medial longitudinal arch mechanics, foot structure (e.g., arch height, ligamentous stiffness) was not assessed. Since the insoles’ effects may differ based on intrinsic foot stiffness or pre-existing arch morphology (e.g., pes planus vs. pes cavus), without quantifying these anatomical factors, the generalizability of our findings across foot types remains uncertain. Future studies should incorporate foot structure assessments (e.g., arch index, 3D scanning, or dynamic arch kinematics) to determine how insole efficacy interacts with individual anatomical variations. Second, this study focused on kinematic and kinetic outcomes but did not examine the interaction between jump performance (e.g., height, power) and biomechanical changes. A comprehensive analysis linking these variables could clarify whether the observed ankle adduction or reduced internal rotation moments translate to functional improvements (e.g., energy return, injury resilience). Finally, the insoles’ material properties (rigid vs. semi-rigid) were standardized, but their interaction with the foot’s natural compliance (e.g., plantar aponeurosis tension) was not quantified. This limits mechanistic interpretations of how extrinsic (insole) and intrinsic (arch) stiffness jointly modulate biomechanics, which shall be investigated in future work.

## 5 Conclusion

This study provides compelling evidence that 3D arch-support insoles significantly modify ankle joint biomechanics during CMJ in a time-dependent manner. Through SPM analysis, we observed distinct temporal effects: during propulsion and flight phases, the insoles primarily influenced joint kinematics by reducing ankle plantarflexion while increasing ankle adduction. While kinetic alterations emerged predominantly during late landing, manifesting as reduced ankle internal rotation moments and decreased ankle anterior and medial forces. These findings carry important implications for insole design, suggesting that optimal performance requires careful consideration of both structural parameters and material properties in relation to specific movement phases. Future investigations should focus on evaluating the long-term effects of these biomechanical adaptations across diverse populations, as well as their potential to mitigate injury risk during dynamic athletic movements.

## Data Availability

The original contributions presented in the study are included in the article/Supplementary Material, further inquiries can be directed to the corresponding author.
