# Supplementary material for: Biomechanical impacts of 3D arch-support insoles on countermovement jumps: a statistical parametric mapping analysis
Source: Front Bioeng Biotechnol. 2025 Aug 26;13:1624892. doi: 10.3389/fbioe.2025.1624892 (PMC12417533; doi:10.3389/fbioe.2025.1624892)
Supplement: Supplementary file 4 [file DataSheet6.pdf]

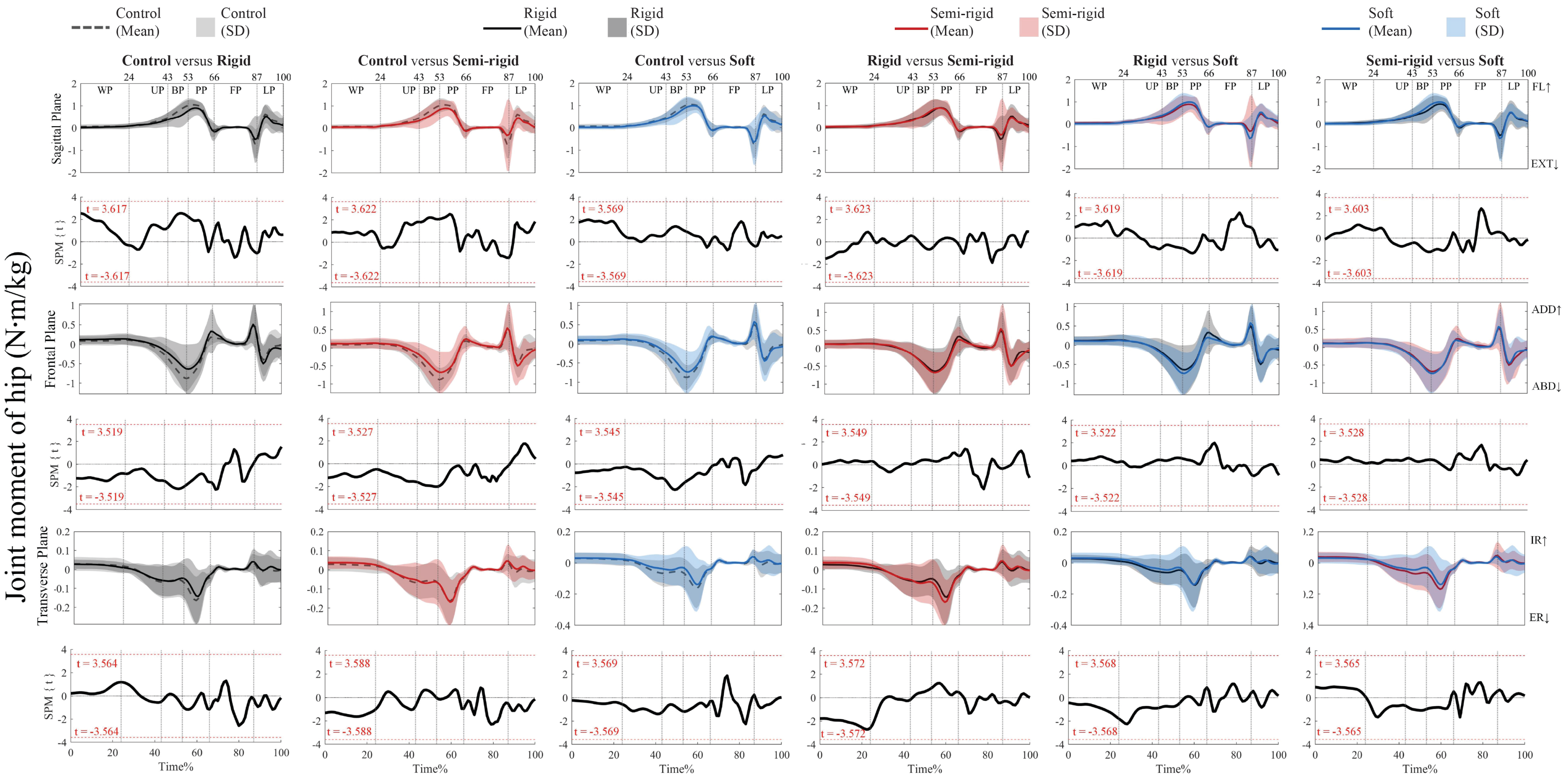

eFigure 6. Mean (SD) patterns for hip joint moments with and without 3D arch-support insoles and time-dependent t-values of SPM (SPM {t}).

Red dashed line represents the critical threshold. WP, weighing phase. UP, unweighting phase. BP, braking phase. PP, propulsion phase. FP, flight phase. LP, landing phase. FL, flexion. EXT, extension. ADD, adduction. ABD, abduction. IR, internal rotation. ER, external rotation.
