# Supplementary material for: Biomechanical impacts of 3D arch-support insoles on countermovement jumps: a statistical parametric mapping analysis
Source: Front Bioeng Biotechnol. 2025 Aug 26;13:1624892. doi: 10.3389/fbioe.2025.1624892 (PMC12417533; doi:10.3389/fbioe.2025.1624892)
Supplement: Supplementary file 6 [file Table1.docx]

**Appendix**

eTable 1. Kinematic and kinetic variables with positive and negative descriptors.

| **Description** | | **Sagittal plane**  **(X-axis)** | | **Frontal plane**  **(Y-axis)** | | **Transverse plane**  **(Z-axis)** | |
| --- | --- | --- | --- | --- | --- | --- | --- |
|  |  | **+ve descriptor** | -**ve**  **descriptor** | **+ve descriptor** | -**ve**  **descriptor** | **+ve descriptor** | -**ve**  **descriptor** |
| Kinematic variable | Ankle angle | Dorsiflexion | Plantarflexion | Adduction | Abduction | Internal rotation | External rotation |
|  | Knee  angle | Flexion | Extension | Varus | Valgus | Internal rotation | External rotation |
|  | Hip  angle | Flexion | Extension | Adduction | Abduction | Internal rotation | External rotation |
| Kinetic variable | Ankle moment | Dorsiflexion | Plantarflexion | Adduction | Abduction | Internal rotation | External rotation |
|  | Knee  moment | Flexion | Extension | Varus | Valgus | Internal rotation | External rotation |
|  | Hip  moment | Flexion | Extension | Adduction | Abduction | Internal rotation | External rotation |
|  | Ankle force | Tension | Compression | Medial | Lateral | Anterior | Posterior |
|  | Knee  force | Anterior | Posterior | Medial | Lateral | Tension | Compression |
|  | Hip  force | Anterior | Posterior | Medial | Lateral | Tension | Compression |
